# Breaking the Silence on Food Risks for Elderly People Living Alone

**DOI:** 10.3390/nu16162655

**Published:** 2024-08-11

**Authors:** Miguel Company-Morales, Lina Casadó-Marín, Araceli Muñoz, Andrés Fontalba-Navas

**Affiliations:** 1 Seron Primary Care Center, Northern Almería Integrated Healthcare Area, 04600 Almeria, Spain; 2 Department of Nursing, Physiotherapy and Medicine, University of Almería, La Cañada, 04120 Almeria, Spain; 3 “Toxic Body” Interdisciplinary Network, Department of Social Anthropology, University of Barcelona, 08001 Barcelona, Spain; linacristina.casado@urv.cat (L.C.-M.); aracelimunoz67@ub.edu (A.M.); andres.fontalba.sspa@juntadeandalucia.es (A.F.-N.); 4 Department of Nursing, Medical Anthropology Research Centre (MARC), University of Rovira i Virgili, 43003 Tarragona, Spain; 5 Medical Anthropology Research Center (MARC), University of Rovira i Virgili, 43005 Tarragona, Spain; 6 Training and Research Unit, School of Social Work, University of Barcelona, 08035 Barcelona, Spain; 7 Research and Innovation Group in Social Work (GRITS), Training and Research Unit, School of Social Work, University of Barcelona, 08035 Barcelona, Spain; 8 Antequera Hospital, Northern Málaga Integrated Healthcare Area, 29200 Malaga, Spain; 9 Department of Public Health and Psychiatry, University of Málaga, 29016 Malaga, Spain

**Keywords:** food, elderly people, malnutrition, silence, loneliness, depression, learned helplessness

## Abstract

(1) Background: Currently, numerous qualitative research studies on food and its influence on health are being conducted. In qualitative research, data are obtained by analyzing participants’ responses. However, silence during conversation has been little studied. The aim of this study was to interpret the silences in the narratives of elderly people living alone about the potential risks of not keeping a healthy diet. (2) Methods: This is a descriptive and interpretative observational study under the qualitative research paradigm following a phenomenological and ethnographic perspective. The study was developed in two phases with people over 65 years old. In the first phase, from June 2021 to January 2022, 90 interviews, 12 life history analyses, 58 food diaries and 51 *free listings* (cultural domain technique) were conducted. In the second phase, from March to June 2022, 3 participatory workshops and 24 *pile sorts* (cultural domain technique) were conducted, as well as 3 focus groups. Only data from participants over 65 years old living alone are analyzed in this paper. The ATLAS-ti (Version 22) qualitative analysis software was used for coding and data analysis. (3) Results: The results show that elderly people living alone would sometimes remain silent during the various conversations conducted within the research. This silence reflects their desire to downplay the risks to their health from not eating well due to their unwanted loneliness. The people participating in our research had chronic health problems, financial insecurity and emotional problems. (4) Conclusions: We concluded that elderly people living alone are unable to maintain a healthy diet because they downplay their risk of malnutrition. This mindset is caused by their loneliness and bolstered by a situation of learned helplessness and social injustice.

## 1. Introduction

The number of elderly people in Spain is steadily increasing, as in other developed countries [1]. Older people have a greater need for protection and are more vulnerable to chronic diseases, while being more dependent in terms of activities that involve daily living skills [2]. The self-care problems of the elderly are aggravated when they live alone, a condition that affects 25% of people over 65 in Spain [3]. The reasons for this aloneness are often the loss of life companions and isolation from their families. Aloneness in the elderly results in fewer social relationships and fewer opportunities to meet other people. Thus, there is a greater probability of becoming isolated at home with a potential risk of behavioral and emotional problems [4]. It is important to note that aloneness in the elderly has a gender component. Women make up the majority of elderly people living alone, as they tend to live longer than men, while also possessing greater independence for self-care and the performance of daily living activities [3].

The early detection of loneliness in the elderly is a current concern to society because of the risks it poses to their health [5]. Different studies show that social isolation fosters an increase in morbidity and psychological problems and is related to a decrease in well-being and health indicators [6]. Primary health care is the most favorable level of health care for the early detection of loneliness. Health care practitioners working in primary care are active in the community and know the context of the patients they see [7]. However, detecting this problem is not a priority for health care services. This fact, together with the silence about the loneliness of elders, makes this problem invisible and unnoticed in self-sufficient older people. Deteriorating health and dependency are often the first signs of the health risks posed by loneliness in older adults [8].

Some authors [2] argue that, in addition to the usual behavioral and physiological problems among the elderly (loss of appetite, altered sense of taste, dental problems), social factors have a significant impact on eating practices among the elderly. Changes in the home, due to the absence of a spouse, siblings or life partners, as well as other life shifts, such as retirement, illness or moving, are key factors in changing eating habits. However, eating is an essential act of life and we now know that it is a fundamental element for successful aging [9].

The aging process typically comes with numerous changes in the body, which generally have a negative impact on the health and lifestyle of the elderly. Among the various factors that influence an elderly person’s state of health, nutrition deserves special attention; maintaining good nutritional status plays a vital role in their quality of life, including physical, mental and social health [10]. Physiological decline related to the quality of food intake is very common among the elderly, often leading to nutritional deficiencies. These deficiencies are major risk factors for certain chronic diseases and age-related health deterioration. Therefore, implementing food-related interventions in elderly patients living alone may be a critical measure [11].

Detecting loneliness at an early stage is not always easy. Many elderly people who live alone do not talk about the emotional problems and physical limitations they suffer due to lack of support. Different authors [12] warn about the need to pay attention to the emotional state of elderly people who live alone because of the impact it has on nutrition. Loneliness is a potential risk for nutrition as it influences food choice and preparation. In many cases, the emotional state of the elderly is more easily perceived by their silence rather than by what they express [13].

Silence is a resource that permeates communication practices. People communicate their ideas, values and beliefs, relying mainly on spoken word and nonverbal language, but also through silence. Silence in the elderly may be an expression of their skepticism that communicating to others about their emotional state will make a difference [14]. Restraint in self-expression, in order not to disturb others, becomes a kind of silence that is difficult to interpret. Jorwoski highlights the importance of interpreting the silence of the subjects’ discourses as a pragmatic reflection of “what is left unsaid” [14]. Authors such as Camargo and Méndez) [15] argue that this pragmatic meaning of silence during conversation has a potentially interpretable intention by those who receive the message. However, the meaning of silence, in order for it not to be diffuse, must always be interpreted in connection to the context and conversational interaction. Thus, along with the relationships that exist between various social norms, silence highlights the interweaving of contexts [15]. Silence is therefore an essential element for the interpretation of discourse.

**Silence about food risks** in the elderly is an issue of great significance, as proper nutrition is crucial to maintaining the health and well-being of the elderly, particularly those living alone. This silence refers to the lack of awareness, discussion and action on nutritional and dietary issues affecting the elderly population. Addressing these risks is critical to preventing malnutrition and food-related diseases, as well as improving the quality of life of older adults, especially those living alone.

### 1.1. Food Risks in the Elderly Living Alone

**Malnutrition:** Malnutrition is a significant food risk for the elderly. It can result from insufficient food intake, malabsorption, chronic illnesses or medications that interfere with adequate nutrition. Malnutrition can lead to weight loss, muscle weakness, fatigue and a weakened immune system, increasing the risk of infections and disease. The available literature has highlighted an unfavorable intake of total fat and saturated fat, sugar, salt and dietary fiber, along with low intake and suboptimal status of key micronutrients such as vitamins D, B2, B12, folate and calcium [16].

**Dehydration:** Dehydration is common among the elderly due to decreased thirst sensation, inadequate fluid intake or diseases and medications that increase fluid loss. Dehydration can lead to confusion, constipation, urinary problems and, in severe cases, life-threatening complications [17].

**Eating difficulties:** Dental problems, chewing or swallowing difficulties (dysphagia), and a diminished sense of taste and smell can make eating challenging for the elderly. This can lead to inadequate intake of essential foods and nutrients [18].

**Chronic disease and medication**: Chronic diseases such as diabetes, heart disease and osteoporosis require special diets and nutritional care. In addition, some medications can affect appetite and nutrient absorption and may have negative interactions with certain foods [19].

**Limited access to healthy foods:** Economic factors, reduced mobility or lack of transportation may limit the elderly’s access to fresh and healthy food, leading to a poor and unbalanced diet. For women, cognitive impairment or being recipients of social support were predictors of malnutrition. Predictors for men were having falls in the past 2 years, hospital admission in the past year and self-reported difficulty climbing stairs [20].

**Commensality:** Commensality, the act of eating together, in addition to nutritional aspects involve elements of a social and symbolic order [21]. When we eat together, we share a table, a sense of hospitality and a bond that we create and reproduce. Eating together is a communal event that cannot be limited to the process of nourishing the body. What happens then to the elderly who eat alone? How does aloneness and loneliness affect their eating practices? What is their perception of the food given to them by food banks or food-aid programs aimed at the elderly?

### 1.2. The Importance of Breaking the Silence

Breaking the silence around these dietary risks involves raising awareness among older people, their families, caregivers and health professionals. Education about proper nutrition, promotion of health-status monitoring and access to health education programs on nutrition are crucial steps to address these challenges [22]. In addition, the living environments of the elderly need to be adapted to facilitate healthy and safe eating, as well as providing support for those who face difficulties in eating or accessing nutritious foods [23].

In conclusion, the silence around food risks among the elderly is a serious problem that requires immediate attention and action. Through education, support and appropriate intervention, it is possible to improve the nutrition and quality of life of the elderly population, ensuring that they can enjoy a healthy and dignified old age. In view of the above, we believe that the contextualized silence in the discursive practice of elderly people living alone should be analyzed for a better understanding of their nutritional reality and early interventions should be implemented to meet their needs. In this paper, we ask ourselves how the loneliness and precariousness in which many elderly people in Spain find themselves impact and pose a risk to their physical and emotional health. We analyze the silences, since what is not said is as important as what is actually said and goes beyond the verbalized discourse. This research stems from an interest in the functions of psychological and normative silences [24] displayed by the elderly in relation to their perceptions of their loneliness and the risks posed to their nutrition and health status. Specifically, the silences interpreted in this study are framed within the narratives provided in interviews, focus groups and food diaries of elderly participants in the Spanish regions of Andalusia, Catalonia and the Valencian Community. In particular, the aim of this research was to interpret the psychological and normative silences in the discourse of elderly participants about their lonely situation. In addition, we also explored possible emotional motivations and guardedness reflected by individual silences about eating practices. Also explored were processes of resistance to social, cultural and contextual conventions as determinants of silence in the assessment of loneliness as a risk for healthy eating.

## 2. Materials and Methods

### 2.1. Study Design

This paper is part of an interdisciplinary research project entitled “Eating matters: Challenges to inclusive, healthy and sustainable food for better aging” (Ref. PID2019-104253RB-C21&C22). This project analyzes aging and social exclusion, with the aim of achieving healthier and fairer food practices in the face of existing social inequalities. Within this context, the project also hopes to foster a transformation of existing social policies.

While the research took a combined quantitative and qualitative approach, this paper is based on the data from the narratives collected through the different qualitative techniques used in the research. It is a qualitative methodological approach which, by taking an interpretative perspective, allowed for an exploration of the actual experiences of these elderly people while including the complexity of the social reality that constitutes their living situations [25].

### 2.2. Study Sample and Setting

The sample selection process was non-probabilistic, intentional and reasoned, taking into account the research objectives. Through qualitative research sampling strategies of intensity and maximum variation, we worked with a heterogeneous sample in terms of gender, age group, and socioeconomic and educational level. Following the inclusion criteria, the study cohort was made up of individuals over 65 years of age who lived in lower to middle income neighborhoods. The exclusion criteria were having difficulties in speaking and/or understanding or not being able to decide about their food practices and/or buy their own food.

The main characteristics of the participants over 65 years of age are described in Table 1 (first phase) and Table 2 (second phase). The main characteristics of the participants over 65 years of age living alone are described in Table 3.

Regarding ethical rules, approval was obtained from the relevant committees and all participants were duly informed of the objectives and methodology of the research. Likewise, anonymity, confidentiality and data protection were ensured at all times and each participant signed a written informed consent form. Names have been omitted in order to ensure the anonymity of the individuals who took part in the research.

Fieldwork for this research was carried out in the Spanish autonomous communities of Catalonia, Valencia and Andalusia, selecting neighborhoods and municipalities on the basis of the research objectives and taking into account the maximum heterogeneity according to social, demographic and environmental criteria. In Andalusia, fieldwork was carried out in the municipalities of Humilladero and Antequera, as well as in the city of Granada. In Catalonia, fieldwork included three neighborhoods in the city of Barcelona, some neighborhoods in the city of Tarragona and several municipalities in the regions of Tarragonés, Terra Alta, Ribera d’Ebre and El Priorat. In the Valencian Community, fieldwork took place in the municipality of Teresa and in some neighborhoods in the city of Valencia.

### 2.3. Data Collection

**Figure 1:**
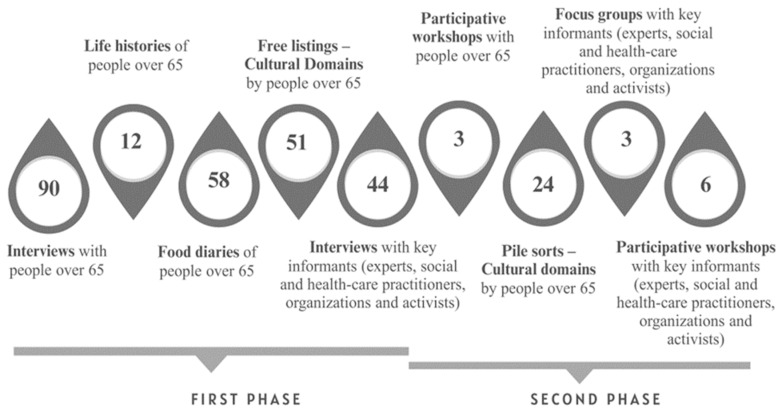
Qualitative data collection techniques used in the research. First and second phases.

Qualitative research took place in two phases (Figure 1). The first phase of the research was carried out from June 2021 to January 2022, including 90 interviews, 12 life history analyses, 58 food diaries and 51 *free listings* (cultural domain technique) with people over 65 years of age. Additionally, another 44 interviews were conducted with key informants (field experts, social and health practitioners, organizations and activists). In the second phase, from March to June 2022, 3 participatory workshops were held with people over 65 years of age and 24 *pile sorts* (cultural domain technique), as well as 3 focus groups and 6 participatory workshops with key informants (field experts, social and health practitioners, organizations and activists). This article only analyzes the data from participants over 65 years of age, and not the narratives of experts, practitioners or activists. It also does not analyze the research data from the two cultural domain techniques, free listings and pile sorts, which have been explored in another paper [26].

### 2.4. Data Analysis and Categorization

The narratives obtained from the interviews, life histories, food diaries and participatory workshops conducted during the research were audio-recorded and subsequently transcribed. These narratives made it possible to explore the experiences of the elderly interviewed within the contexts related to food, in order to analyze silence about food risks among this age group.

The narratives were analyzed and systematized with the support of the qualitative analysis software ATLAS-ti (Version 22; ATLAS-ti Scientific Software Development GmbH; Berlin, Germany, 2022), following Grounded Theory strategies [27,28]. Thematic categories linked to the research objectives were identified and these categories were grouped by segmenting the narratives by units of meaning and assigning codes to them in order to group them into an interpretative framework. In order to visualize and represent the relationships between these codes, semantic networks and diagrams were generated. Discursive silence by participants was represented in the transcripts with three dots in parentheses “(…)”.

## 3. Results

### 3.1. Epistemic and Psychological Silence

**Figure 2:**
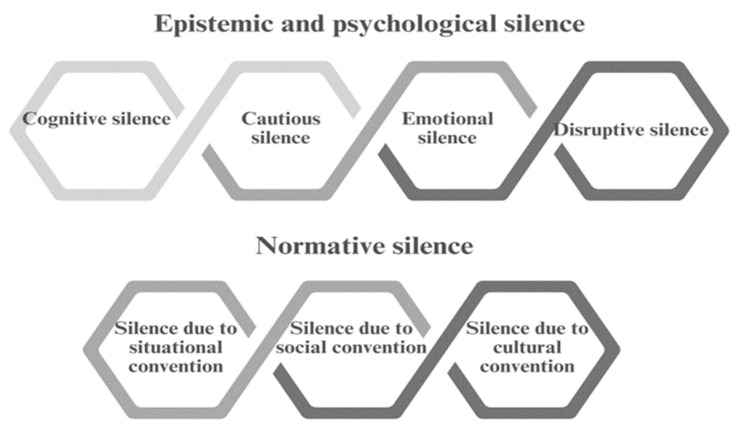
Epistemic and psychological silence, and normative silence. Own elaboration based on data from “La Pragmática del silencio en la conversación en español. Propuesta taxonómica a partir de conversaciones coloquiales” by L. Camargo and B. Méndez [24].

First, we grouped narratives of elderly people that included silences related to an affective, expressive and cognitive dimension. These silences can be interpreted in the context of our fieldwork as emotional containment or acts of hesitation while participants were sharing their thoughts. Epistemic and psychological silences involve potential pragmatic functions that include, in particular, the so-called “cognitive or reflective”, “cautious”, “emotional” and “disruptive or resisting” functions (Figure 2).

#### 3.1.1. Cognitive Silence

Some elderly subjects in our study remained silent in a reflective attitude and hesitated about how they could put certain thoughts into words. This silence allowed participants to mentally organize their discourse about potential environmental risks and food uncertainties in their area of residence.

A female subject, with no primary education, lives alone on an income of less than EUR 1200 per month. She states that she has been receiving food assistance provided by the local government since 2018. She has seven children and when her children were still young she left home upon learning of infidelity by her husband. Her ex-husband did not give her any money for the children and she has had to work and apply for child support ever since then. Although her children are now grown, she receives small subsidies that make it easier for her to make ends meet:

“Here they help me every (…) they used to give it every week, now they give it every fortnight… if they give you potatoes, then that’s something I don’t have to buy myself, if they give you rice, I don’t have to buy it myself, if they give soup, the same. They give you a bottle of oil every two weeks, well, a bottle of oil, you see what I mean? (…) and we’re managing like that.”.

(Woman, 74 years old, Catalonia)

Presently, she is accompanied to collect her food, as she has diabetes and sometimes gets light-headed and has suffered some falls “because of the blood sugar”. Regarding the food she is given, she also says: “They give me a carton of milk, they give me a liter of oil. If there are potatoes, they give me potatoes, if there is chicken they give me a half chicken (…) And if they give fruit, they give me… one… little bag with fruit. I have no complaints”.

Below is an example of how a person living alone expressed his views on the provision of food to the elderly by various agencies, without appraising the situation of loneliness:

“There are people, for instance, who don’t have an elevator in their homes and going out is a big deal, or (…) they are lonely and tend to somewhat isolate themselves. Then, sometimes, these people, through other programs, which are not the ‘Food at Home’ one [similar to Meals on Wheels], maybe the public administration does have (…)”.

(Man, 67 years old, Valencia)

Here, we can see how this participant falls into cognitive silence in order to organize his speech in a coherent way, demonstrating how significant it is for him that some elderly people living alone cannot travel to the place where they have to pick up the food provided by the aid program.

#### 3.1.2. Cautious Silence

At certain times, elderly participants would be cautious or withhold some of their views.

A widowed woman living alone with an income of EUR 1200–1500 a month had to go to a Caritas [a Catholic charity] food bank for a while. Her husband was a heavy drinker and squandered all their household income. She reports that this situation was greatly distressing for her, until she decided to leave him when she was older and left her home to work as a live-in caregiver for the elderly:

“It’s a bit of a long story (…) my husband was a lot of trouble. He gave me a lot of grief. I strived to earn money, but then it would all just vanish. Even my wedding ring… he went and sold it. I mean, when I couldn’t work anymore (…) I deteriorated a lot, I was, I was (…) well, in really bad shape. And then my GP told me: ‘If you don’t leave, it’s going to be the end of you. You will die, you won’t be able to go on’. So I spent two or three years away from home. I was looking after older people. I was living, I mean, I was a live-in caregiver (…)”.

(Woman, 82 years old, Tarragona)

Loneliness was not felt by this participant during her time as a live-in domestic worker but this makes her guarded about her current loneliness. This is probably related to her mixed feelings about the liberation she felt upon escaping the hard life imposed on her by her ex-husband, on the one hand, and the lack of company and support at present in her old age, on the other hand.

#### 3.1.3. Emotional Silence

Some participants seemed disconcerted while talking and appeared to become emotional when addressing the topic of loneliness, a topic that made it impossible for them to continue expressing themselves verbally.

A widowed woman living alone became emotional when recalling the early deaths of her children. This made her fall into depression, a condition that she acknowledges as a reason for her health worsening, along with her loneliness.

“Well, I suffered from depression because I lost my children, one at age 43 and the other at age 39. My younger daughter left a 6 year old girl. I carry this baggage with me constantly and fell into a depression. And I think I started to lose (…) my health a bit ever since then.”.

(Woman, 84 years old, Valencia)

A divorced man with no primary education is living alone. He has an income of EUR 1200–1500 euros. When asked about the support he receives, he reported the following:

“I have a lot of problems now because I can’t make ends meet. That’s why I went to Social Services. I was drinking for two months, and here, if you keep drinking for three months, they kick you out. I saw that I wasn’t going to make it, three months and out (…) Then, finding myself in the street at 77, I was feeling, hmm, I’m redundant, I’m redundant in life (…) and I got it into my head that ??? and mind you, I’m still in this situation, but anyway, I’m more hopeful (…) lucky to have Vanesa… and then I say, I have to keep going. (…) [Social Services] called me for a second, they asked if I needed anything and I said I didn’t. I do need help. But I’m not gonna come here for them to sort out my life. They offered a solution, I thanked them. They told me that they might take me to the food bank. Yes, because I can’t go on my own, I have this problem, I have this… There must be people who are worse off than me, you know? And since they did this great favor to me (…) one could say they gave me back my life, because I was ready to give up. Between my sister-in-law and them… Between my sister-in-law and Vanesa, it’s my lifeguard (…)”.

(Man, 78 years old, Barcelona)

#### 3.1.4. Disruptive Silence

Other times, participants remained silent during their intervention as a sign of disagreement or rebellion against a line of argumentation accepted by the majority of the group or disagreement with the researchers.

A participant, separated and living alone, told us about the ‘Food at Home’ program available to some elderly people who live alone and have mobility problems.

“There is a program, ‘Food at Home’ (…) which many cities also have, especially larger ones. Well, these ‘Food at Home’ folks, they come to your house, deliver the food and that’s that. And then, as far as I know, I don’t know if it has changed, maybe it’s changed recently and I’m not up to date, it doesn’t work on weekends, and of course (…) the result is… What’s these people’s situation? They are people who have mobility problems because of architectural barriers, don’t have elevators in their buildings and going out is a huge deal, or else they’re alone and somewhat isolated (…)”.

(Man, 69 years old, Valencia)

### 3.2. Normative Silence

This kind of silence is heavily ruled by situational, social and cultural convention. It is based on a set of norms and rules that a community accepts and practices for the harmonious development of its interactions.

A separated man living alone says that financial aid such as a government payment card is essential, since it affords him greater autonomy in choosing his food.

“A prepaid card for economic aid is fundamental (…) I do believe that politicians should conduct a more global review of the value of a prepaid card. It helps a person to enjoy better health… It’s not about the money. The person making purchases should have to show all the receipts of what they’ve spent. They must do that. They should be able to show what they’re spending this money on-show that you didn’t buy a crate of beer, or two bottles… That you bought milk. This is the person’s responsibility. But, yes, I do think that prepaid cards and financial aid are essential for families, as they can then choose what they think is good for them. (…) That makes them independent … being able to choose. And then… for instance, my son is allergic. He has asthma. So there are foods that he cannot eat (…) If I have a prepaid card, I can go to the market and buy fresh and healthy food. If I don’t have a prepaid card, I have to go to the social center and there they give me some canned tomatoes, and this and that, and I take it home. The difference is huge. So huge that one thing kills you and the other doesn’t.”.

(Man, 66 years old, Barcelona)

A woman living alone highlights the importance of vegetables in her diet and the potential interactions with the medication she takes:

“I think all kinds of vegetables are healthy. [Interviewer]: Of course. [Participant]: Vegetables and those… […] I eat a lot of tomatoes. Fresh, yes, I take them out of the fridge, split them, and add some salt and oil-I love them. Tomato, garlic, I eat all of that. Now, one thing I really really love is chard. I could eat chard every day. But I can’t eat chard because I’m taking Sinthrome…”.

(Woman, 83 years old, Barcelona)

#### 3.2.1. Silence Due to Situational Convention

Different participants would sometimes keep silent because of context, particularly in focus groups.

An elderly man living alone reports that he has used food banks on occasion. When asked specifically about the type of diet and access to food he has, he answered the following:

“[Interviewer]: On occasion you’ve had to get food from the food bank. So they could give you a hand, right? [Participant]: Well, yes, there you go, there you go. [I]: Okay. What about fruit? Do you eat fruit? [P]: Fruit, not much. As there is… there is… there is little. [I]: The food, let’s say, what we eat, is somewhat related to the money we have, isn’t it? [P]: So it is, so it is. [I]: Okay, and fish, how long has it been since you last ate fish? [P]: Hmm, I haven’t eaten fish since… I… I… I don’t remember. [I]: You don’t remember when you last ate fish? [P]: I don’t remember that.”.

(Man, 71 years old, Humilladero-Málaga)

#### 3.2.2. Silence Due to Social Convention

Sometimes, participant’s silences were caused by the perceived unbalanced relationship with the facilitators/researchers. The imbalance stemmed from the participants’ perception of researchers as health and environmental experts.

A male participant who receives a pension of between EUR 800 and EUR 1200 per month, when asked about the types of food he buys and their prices, reports the following:

“Oh! And […,] get that, in Lidl (a supermarket chain) just some […] three, about three months ago or even less, I don’t know, a dozen eggs cost EUR 1.45 and now it’s EUR 1.75. You see? [subject chuckles] And it’s a […] a […] a […] a big place, where many people go and they raise prices without any consideration to anybody, only […] and don’t get me going on electricity, well, that’s just plain outrageous. […] Then you need gas, you need electricity and gas, and water to wash yourself [subject chuckles], to take showers. You need everything. [I]: Do you think that maybe the government should provide some kind of aid to elderly people specifically for food, for cooking, for… [P]: Look here… When they said that people […] vulnerable people […], the-the-the government said they would give something towards electricity […] that we would get a discount, we would get […] and we filled out all the paperwork, we filled it all out, we did it all, and then they refused it. We did it again. Refused, and refused again and again […] Aid? None at all.”.

(Man, 81 years old, Barcelona)

The issue of loneliness also came up in an interview with a woman with an income of less than EUR 800 per month.

“Well, we cannot take away the age we are. Nor loneliness.”.

(Woman, 85 years old, Humilladero-Málaga)

#### 3.2.3. Silence Due to Cultural Convention

Our participants’ narratives often showed culturally motivated silences and expressed local clichés and taboos about the potential dietary risks of living alone.

A female participant who lives alone is a widow with an income of less than EUR 800 a month. When we asked her: Do you live alone?, she said the following:

“Alone… yes. That’s why I have remote senior assistance. To get it, you have to be low income and living alone.”.

(Woman, 77 years old, Valencia)

Another participant, with a primary school education and an income under EUR 800 per month, had to resort to the food bank because of her situation. Due to mobility problems, two people had to pick up her food allotment for her:

“Until two months ago, I had to feed myself with help from the food bank, but that is (…) always the same parcel; they give me two cartons of milk every 15 days. They have to last for 15 days, and that’s not even enough to get started, because that’s what I always have for breakfast. And little else (…) I couldn’t go, so two friends went to pick it up and bring it to me. I couldn’t go myself.”.

(Woman, 65 years old, Granada)

While she views resorting to the food bank as a necessity, when asked specifically about soup kitchens and the possibility of going to one, she says she would never go there, no matter how bad off she was:

“I don’t know, that’s something that (…) I didn’t want. For myself, no way. From what I have seen, it’s not like, or maybe it is, but it’s not like unemployed lawyers go to soup kitchens. The lines I’ve seen, because I’ve seen what I’ve seen, and me with those people, they’re as worthy as myself, but I can’t see myself together with them. I’m not like them. [I]: What are these people like? [P]: There are drug addicts, drunkards, people, very good people, who live their own way, as they like (…) but me (…) I have seen many kinds of people in food bank lines (…) but a soup kitchen is different. I mean, I have been there, I can’t say I haven’t. I have gone to get food, because I have to have something at home and I need to feed myself. But there is a difference. Let’s see, nowadays everybody has to be willing to stand in line at the food bank because there’s no other choice, so I (…) well, I… maybe you’ll say, when you’re in need, yes, but (…) but I don’t know, one’s principles, one’s (…) dignity (…) embarrassment (…) it hurts a lot, and with the life I’ve built for myself (…) this hurts (…) it hurts a lot!”.

(Woman, 65 years old, Granada)

## 4. Discussion

Psychological and epistemic silences are essential in our study because they show the cognitive and emotional state of the elderly while interacting with others. Normative silences offer a social orientation to the work carried out in this research, as these silences are motivated by situational, social and cultural convention. Méndez (2016) [29] argues that silence is an element present in communication that signifies and is used to signify. The main communicative purpose of silence is to transmit information. The psychological and normative silences [24] shown by the participants during the interviews, focus groups and ethnographies have been an important source of information. The contextualization of this information, in the residences of the elderly participants, has made it possible to draw inferences about their loneliness and the potential risks it poses to their nutrition status.

Our research shows, in line with other studies [30,31,32], that psychological and epistemic silences [24] were the most numerous after interpretative analysis of elderly participants’ narratives. Older people displayed psychological and epistemic silences reflecting their emotions, hesitations and degree of interest in talking about the influence of loneliness on proper nutrition. In the participant interviews, it was possible to observe how they often maintained silence in a way that demonstrated the kind of reflection that allowed them to mentally organize their narrative. The elderly participants in our study showed a reticence to bothering their children, relatives or friends, so they appeared to not want to say anything that was not “socially correct”. This particularity was more noticeable in focus groups, due to the fact that the participants did not know each other [33], thus increasing the instances where older people were reluctant to express their feeling of loneliness.

In the three Spanish regions where our work was carried out, the population over 65 years of age is higher than the national average, and many of these people live on their own [34]. The particular motivation for our work arose from the observation during fieldwork of signs of unwanted aloneness in some of the elderly participants in our research. This blatant reality emerged during the development of the work and had not been included in the original design. Authors such as Luna and Pinto (2021) [5] argue that aloneness/loneliness is a complex concept, which is difficult to express, and that it is part of the subjective world of each individual. This makes its analysis and approach a challenge, given that when we talk about loneliness we are not only referring to the fact of being or living alone but also to negative connotations of being lonely such as isolation or a feeling of abandonment. Therefore, when we talk about loneliness, we talk about feelings, about feeling alone. One participant in our study expressed this feeling of loneliness as something inevitable: “We cannot take away the age we are (…) nor loneliness”. Elderly people stated on several occasions that loneliness was inevitable when a person gets older and has no partner or family. The psychological and epistemic silences displayed during the narratives support this idea. However, the authors of this study are aware that we can also speak of a kind of wanted aloneness, solitude. As one participant remarked: “I can tell you that I am never lonely, because I get along well with everyone, and besides, I am happy at home”. It is therefore necessary to point out that this study focused on the undesired aloneness (loneliness) of the elderly.

Some research participants displayed emotional silences when their narratives addressed the topic of depression and how it affected their eating practices and health status. Various studies have shown that loneliness is exacerbated by depression, illness, lack of contact with family and friends, and death of a spouse [10,35,36]. We also observed that some participants fell into emotional silences when talking about how their financial problems affected their eating habits—a fact that caused them not to ask for help and to lead even more lonely lives. These results are in line with other studies showing how economic difficulties in the elderly who live alone impact their food practices, quality of life and health [2].

Some elderly people showed disruptive silences during their interventions when talking about some home-delivered meal programs. Specifically, one participant who lives in Valencia was critical of these programs and believed that these home-delivery programs are limited to leaving food at the door, neglecting to monitor the food intake of the elderly and their state of loneliness and isolation. Various authors have reflected on the usefulness of these home-delivery meal programs provided by some local governments in large Spanish cities and have found that the quality of the food and delivery schedules are often inadequate (some only provide one meal per day and others do not cover weekends) [5].

We know from different studies that the feeling of loneliness is significantly associated with malnutrition among the elderly [10,35,36,37,38]. Therefore, we can argue that older people who feel lonely are at high risk of malnutrition. Similarly, loneliness has an impact on malnutrition by limiting the functional ability to perform activities of daily life such as shopping, cooking, and eating. We can also argue that loneliness among the elderly in our study is a gender issue, as women make up the majority of elderly people living alone. However, older people do not always express these feelings and remain silent, and they increasingly subject themselves to isolation and helplessness. They do not ask for help, thinking that their loneliness has no solution, as expressed by one of the participants in our study.

This behavior is what Martin Seligman [39] called “learned helplessness—a reaction of giving up and not fighting as a consequence of believing that whatever you do, things will not get better” [39]. Some of our study participants believe that it is impossible to get rid of this feeling of loneliness and report emotional problems and depressive states (also observed by other authors) [35], demonstrating typical behaviors of learned helplessness. According to Seligman [40,41], learned helplessness is displayed by people who psychologically tend not to defend themselves from risky situations. This behavior possibly manifests because at some point they tried to defend themselves, to ask for help, and it made no difference to their lives. In the face of constant refusal, they took on an attitude of not acting or defending themselves because they believed that change would not occur. This is similar to what some of the elderly people express in their narratives. They feel incapable of facing their loneliness with self-sufficiency, a state of being that increasingly reflects back to their poor nutrition due to an inability to navigate the tasks of daily life.

Normative silence was another subject of study [24]. In this case, older people displayed silences conditioned by their living and social context. In focus groups, there were more instances of silence than in individual interviews and ethnographies, a fact that supports the findings of other studies [32]. Social convention also promoted silence due to the perceived unbalanced relationship between the elderly and the researchers regarding knowledge of the subject of study. However, the most frequent normative silences were related to cultural reasons and “taboo topics” [7,22,36,37]. Some participants in our research displayed silence when addressing the “taboo” topic of having to beg for food at food banks. These silences were linked to the socially generated attitude regarding people who beg for food. In our society, an image has been created that people who ask for help in order to be able to eat are people in extreme poverty or living in the street. However, it is becoming increasingly common for people who have a salary or a retirement pension to have to resort to food assistance [42].

Based on the narratives of our research and in line with other authors [43,44], we can assert that the number of elderly people who need help to maintain a healthy diet is on the rise. Limited economic resources [45], social problems such as loneliness [36], or health and autonomy problems in the elderly [20,35] are determinants that hinder proper nutrition among the elderly [26].

## 5. Conclusions

In recent years, the increase in life expectancy, the aging of the population and social changes have led to an increase in the number of elderly people living alone. Although the feeling of loneliness does not always occur in people living alone, it is true that unwanted loneliness is more frequently felt among older people who do not share their home with anyone [4]. It is more frequent for women to be lonely because they live longer and are usually younger than their spouses [46], as we have found in this research.

Limited functional capacity in the elderly affects mobility and/or the senses, making it difficult to carry out the tasks of daily life. In our study, we have found that for elderly people living alone, limited functional capacity is a significant obstacle to maintaining a healthy diet and healthy aging. Limited functional capacity means that the elderly person cannot go out shopping and has difficulty cooking, a situation that fosters skipping meals due to reluctance or demoralization. Thus, these people find it more difficult to maintain a proper diet, which in turn leads to malnutrition.

The analysis of the psychological and normative silences produced in the narratives of participants living alone shows that most of the silences are an expression of self-consciousness and an underestimation of the risk of malnutrition. Similarly, they feel resigned to their state of loneliness, which evokes a situation of learned helplessness that prevents them from asking for help or alerting others about the difficulties they experience in carrying out the tasks of daily life.

We can conclude that loneliness can have negative consequences on the health of the elderly, fostering inadequate nutrition that can potentially lead to malnutrition. Similarly, loneliness and lack of communication pose a potential risk for depression in the elderly. It would be advisable for health care and social agencies to address loneliness in the elderly through early detection and deal with this as a public health issue. Most elderly people tend to keep silent about their difficulties and do not want to be a nuisance to others. Therefore, it is important to help these people living alone to break the silence about the risks of inadequate nutrition. It should be a matter of social justice that older people living alone be integrated into different groups to encourage their participation. Society should promote a feeling of belonging and attachment among our elderly people, help them in communicating their problems, and improve their state of loneliness so that they can experience healthy and dignified aging.

This study provides a valuable and introductory study on precautionary attitudes and the effects of loneliness of the elderly’s nutrition. As a limitation, in this research, we want to highlight that the influences that the immediate context and social agents (family, friends, health care professionals, etc.) have on the narratives and silences of the elderly have not been studied in depth. In the way, the results that emerge from this research should be considered as an example for future research that studies the influence of the close context of the elderly in the interpretation and assessment of the impact of loneliness on the nutrition and health of the elderly.

## Figures and Tables

**Table 1:** Sociodemographic characteristics of participants over 65 years of age by region. First phase.

	Andalusia (*n* = 4)	Catalonia (*n* = 11)	Valencian Community (*n* = 9)	Total (*n* = 24)
**Gender**				
Male	2 (50%)	2 (18%)	0 (0%)	4 (17%)
Female	2 (50%)	9 (82%)	9 (100%)	20 (83%)
**Age**				
<75 years	2 (50%)	7 (64%)	5 (56%)	14 (58%)
≥75 years	2 (50%)	4 (36%)	4 (44%)	10 (42%)
**Place of residence**				
Rural	4 (100%)	0 (0%)	9 (100%)	13 (54%)
Urban	0 (0%)	11 (100%)	0 (0%)	11 (46%)

**Table 2:** Sociodemographic characteristics of participants over 65 years of age by region. Second phase.

	Andalusia (*n* = 33)	Catalonia (*n* = 36)	Valencian Community (*n* = 33)	Total (*n* = 102)
**Gender**				
Male	15 (45%)	14 (39%)	6 (18%)	35 (34%)
Female	18 (55%)	22 (61%)	27 (82%)	67 (66%)
**Age**				
≤64 years	3 (9%)	0 (0%)	1 (3%)	4 (4%)
65–69 years	3 (9%)	4 (11%)	7 (21%)	14 (14%)
70–79 years	19 (58%)	18 (50%)	17 (52%)	54 (53%)
≥80 years	8 (24%)	14 (39%)	8 (24%)	30 (29%)
**Place of residence**				
Rural	18 (55%)	17 (47%)	18 (71%)	53 (52%)
Urban	15 (45%)	19 (53%)	15 (29%)	49 (48%)
**Living alone**				
Yes	12 (36%)	18 (50%)	9 (71%)	39 (38%)
No	21 (64%)	18 (50%)	24 (29%)	63 (62%)

**Table 3:** Sociodemographic characteristics of participants over 65 years of age living alone.

	Andalusia (*n* = 12)	Catalonia (*n* = 18)	Valencian Community (*n* = 9)	Total (*n* = 39)
**Gender**				
Male	3 (25%)	5 (28%)	1 (11%)	9 (23%)
Female	9 (75%)	13 (72%)	8 (89%)	30 (77%)
**Age**				
65–69 years	1 (0.8%)	1 (0.5%)	3 (33%)	5 (13%)
70–79 years	6 (50%)	8 (49.5%)	4 (45%)	18 (46%)
≥80 years	5 (49.2%)	9 (50%)	2 (22%)	16 (41%)
**Education Level**				
Primary	3 (25%)	4 (22%)	4 (45%)	11(28%)
Secondary	2 (16.5%)	4 (22%)	1 (11%)	7 (18%)
Higher	2 (16.5%)	3 (17%)	1 (11%)	6 (16%)
Without Studies	5 (42%)	7 (39%)	3 (33%)	15 (38%)
**Marital Status**				
Widow/ed	7 (58%)	11 (60%)	5 (56%)	23 (59%)
Single	1 (0.8%)	2 (12%)	1 (11%)	4 (10%)
Divorced	4 (41.2%)	5 (28%)	3 (33%)	12 (31%)

## Data Availability

The data that have been used are confidential.
